# Revisiting Incomplete Tissue‐Level Reperfusion Following Successful Thrombectomy for Acute Ischemic Stroke

**DOI:** 10.1002/ana.78142

**Published:** 2026-01-26

**Authors:** Yue Qiao, Adrien Ter Schiphorst, Yi Xu, Jean‐Claude Baron, Wenbo Zhao

**Affiliations:** ^1^ Department of Neurology Xuanwu Hospital, Capital Medical University Beijing People's Republic of China; ^2^ Department of Neurology CHRU Gui de Chauliac Montpellier France; ^3^ Department of Neurology GHU Paris Psychiatrie et Neurosciences, Inserm U1266, Université Paris Cité Paris France; ^4^ Stroke Research Group, Department of Clinical Neurosciences University of Cambridge Cambridge UK

## Abstract

Among patients with acute ischemic stroke achieving successful large vessel recanalization (defined as expanded Thrombolysis in Cerebral Infarction [eTICI ≥2b]), incomplete tissue‐level reperfusion, distinct from visually identifiable distal occlusion on digital‐subtraction angiography, remains a significant challenge. Persistent tissue‐level hypoperfusion, identified on post‐thrombectomy perfusion imaging, involves complex pathophysiology comprising 2 primary mechanisms: territorial hypoperfusion, stemming from distal emboli that often manifest after eTICI 2b to 2c or a posteriori with eTICI 3 recanalization, obstructing small distal arterial branches and forming wedge‐shaped patterns; and hypoperfusion within the ischemic core, potentially representing capillary no‐reflow, a phenomenon of microvascular perfusion failure despite successful macrovascular recanalization well described in the preclinical literature. Such pathophysiological differences have driven inconsistent designations, causing reported incomplete tissue‐level reperfusion rate to vary widely (0–42.5%) even in angiographically complete (eTICI 3) recanalization. Further clouding the scene, territorial hypoperfusion may spontaneously reverse within 24 hours (termed “delayed reperfusion”), yet a distinct delayed hypoperfusion can affect necrotic tissue regardless of no‐reflow. Whereas persistent hypoperfusion is significantly associated with functional outcomes, the functional impact of true no‐reflow remains unclear. Recent positive randomized controlled trials (RCTs) of intra‐arterial thrombolysis after successful recanalization offer a promising therapeutic strategy. However, these trials lacked perfusion imaging, whereas the larger functional benefit in patients achieving eTICI 2b suggests intra‐arterial thrombolysis likely acted on distal emboli rather than true microvascular dysfunction. Regarding microvascular dysfunction, preclinical findings highlight potential therapeutic strategies such as targeting pericyte constriction or inflammatory responses, that warrant clinical translation. This review synthesizes current evidence on the mechanisms, assessment methods, and therapeutic strategies for addressing incomplete tissue‐level reperfusion following thrombectomy. Further research is warranted to establish standardized definitions, develop targeted therapies for both territorial hypoperfusion and true no‐reflow, and translate promising preclinical findings into effective clinical interventions. ANN NEUROL 2026;99:668–683

## Graphical abstract

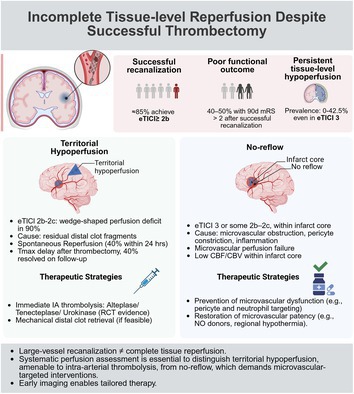

Timely recanalization through endovascular thrombectomy (EVT) has been demonstrated as first‐line treatment for acute ischemic stroke (AIS) due to large vessel occlusion (LVO), significantly improving functional outcomes.^1^ However, a substantial gap persists between successful recanalization and optimal functional recovery relative to expected outcome. Nearly half of EVT‐treated patients do not regain functional independence despite successful recanalization,^2^ a scenario sometimes referred to as “futile recanalization,” although this terminology is problematic because clinical improvement that results in outcomes other than modified Rankin Scale 0 to 2 is not considered. Early and complete reperfusion remains a critical determinant of functional outcomes in patients with AIS,^3^, ^4^ with successful endovascular reperfusion widely recognized in the field as an expanded Thrombolysis in Cerebral Infarction (eTICI) grade ≥ 2b, indicating more than 50% reperfusion of the affected territory. However, substantial heterogeneity exists within the eTICI 2b category, spanning reperfusion extents from 51 to 89%, and accumulating evidence emphasizes that patients achieving eTICI 2b exhibit significantly worse 90‐day functional outcomes compared with those achieving near‐complete and complete recanalization (eTICI 2c and 3, respectively).^5^, ^6^, ^7^

Persistent cerebral tissue‐level hypoperfusion on post‐EVT perfusion imaging despite successful proximal large‐vessel recanalization is a frequent and clinically significant phenomenon.^8^, ^9^, ^10^ The terminology “tissue‐level hypoperfusion” used in this review aims to distinguish reduced nutritional cerebral blood flow (CBF; assessed using validated perfusion tracers) from TICI grading, which is used by neuro‐interventionalists to define degrees of “reperfusion” on digital subtraction angiography (DSA) but assesses arterial branches patency only (to be defined below as “recanalization”). Thus, patients rated as eTICI 3 (even after central core reading to detect distal arterial occlusions) can still exhibit tissue‐level hypoperfusion on post‐thrombectomy imaging, sometimes leading to the detection of initially missed distal emboli (and thus to their downgrading to TICI 2b or 2c).^11^ The reliability and validity of eTICI scoring have substantial variability and are potentially causes for over calling successful recanalization, compounding the discrepancies with incomplete reperfusion on follow‐up perfusion imaging.^12^ In patients not achieving complete reperfusion (eTICI < 3), early post‐thrombectomy perfusion assessment frequently reveals wedge‐shaped (territorial) hypoperfusion indicative of distal vessel occlusions, and immediate flat‐panel computed tomography (CT) perfusion imaging demonstrates hypoperfusion in virtually all patients with eTICI 2b.^11^ In addition, recent studies have shown that incomplete tissue‐level reperfusion in patients with incomplete recanalization persists at 24 hours post‐intervention in approximately 60% of patients, and that persistent hypoperfusion is correlated with poorer clinical outcomes.^13^

Apart from distal occlusions, incomplete tissue‐level reperfusion has been observed in up to 15 to 25% of patients achieving near‐complete or complete reperfusion (eTICI 2c or 3).^14^, ^15^ Contributing processes in tissue‐level hypoperfusion include the “no‐reflow phenomenon,” whereby the previously severely hypoperfused brain tissue fails to demonstrate capillary reperfusion despite successful recanalization of the primary occlusion, due to microvascular failure.^16^, ^17^

Although achieving recanalization of the proximal large vessel is an essential step for patients with LVO‐AIS, tissue‐level cerebral hypoperfusion ultimately impacts functional outcomes, and some studies have found hypoperfusion to be a better predictor than recanalization grade.^14^, ^18^, ^19^ These findings highlight the need for adjunctive therapies beyond EVT. Accordingly, several recent randomized controlled trials (RCTs) have evaluated the effect of intra‐arterial administration of thrombolytic agents as an adjunctive therapy at the end of the endovascular procedure on 3‐month functional outcome.^20^, ^21^, ^22^, ^23^ Although the results of these RCTs were variable (neutral or positive), a meta‐analysis suggested overall clear benefit across trials, particularly in the eTICI 2b category, suggesting the benefit occurs via lysis of distal emboli rather than no‐reflow.^24^ This uncertainty underscores the need for a deeper understanding of the underlying mechanisms contributing to incomplete tissue‐level reperfusion despite angiographic recanalization. Although several previous reviews addressed hypoperfusion despite recanalization,^17^, ^25^, ^26^, ^27^ major breakthroughs in this rapidly evolving field have emerged since they were published, radically changing our understanding of this phenomenon. Notably, they generally did not distinguish territorial hypoperfusion from no‐reflow, which may require different therapeutic approaches as suggested by recently published clinical trial evidence. In the present review we aim to integrate recent evidence from both observational studies and randomized trials to systematically distinguish these different subtypes of incomplete tissue‐level reperfusion after successful thrombectomy.

The present narrative review aims to synthesize current evidence regarding incomplete tissue‐level reperfusion following successful recanalization (eTICI ≥2b), and to distinguish territorial hypoperfusion caused by distal emboli from tissue‐level hypoperfusion potentially linked to no‐reflow within established infarcts. Furthermore, we will examine therapeutic strategies for incomplete tissue‐level reperfusion, highlighting promising preclinical findings.

## Distinguishing the Main Subtypes of Incomplete Tissue‐Level Reperfusion

**FIGURE 1:**
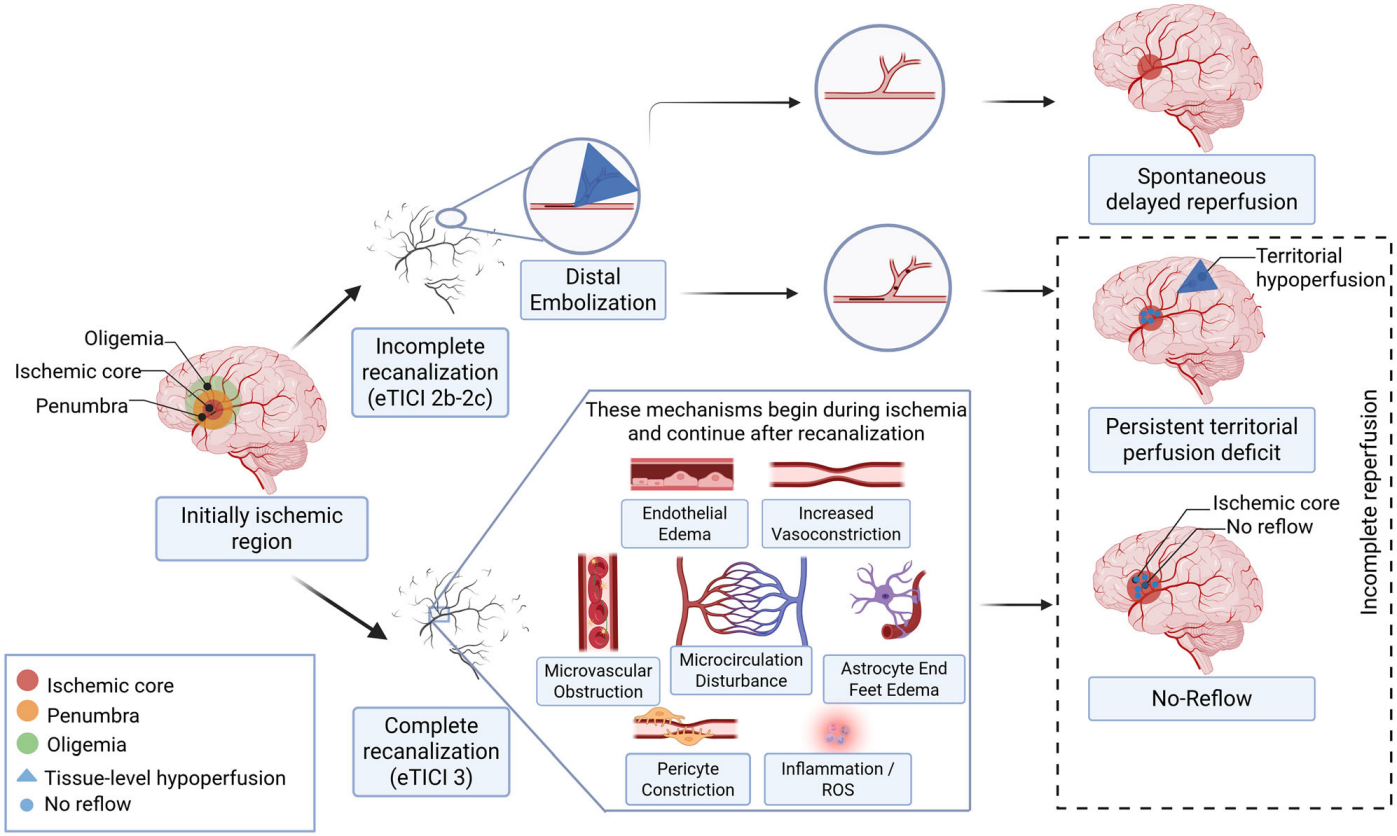
Distinguishing the main subtypes of incomplete tissue‐level reperfusion. Despite successful recanalization, the initially ischemic vascular territory can display persistent tissue‐level hypoperfusion according to 2 main pathways, broadly depending on recanalization status. In case of incomplete recanalization (eTICI 2b–2c), distal embolization prevails, leading to territorial hypoperfusion that may either spontaneously reperfuse or persist, in the latter case potentially causing newly infarcted areas. In case of complete recanalization (eTICI 3), the no‐reflow phenomenon (shown here as heterogeneous, but can also be uniform) may affect the established ischemic core as a result of microvascular obstruction/dysfunction, through multiple potential mechanisms illustrated in the central box. Note that no‐reflow can also occur in patients with incomplete recanalization (eTICI 2b–2c), involving the pre‐thrombectomy ischemic core. Both territorial hypoperfusion and no‐reflow may co‐exist and contribute to incomplete post‐thrombectomy tissue‐level reperfusion, negatively impacting functional outcomes despite successful macrovascular recanalization. ROS = reactive oxygen species; eTICI = expanded thrombolysis in cerebral infarction. [Color figure can be viewed at www.annalsofneurology.org]

As just mentioned, persistent tissue‐level hypoperfusion following successful thrombectomy‐induced recanalization of proximal large vessels can result from various mechanisms, which are paramount to distinguish as they might respond to entirely different interventions (Fig 1). Importantly, they lead to distinct patterns of tissue hypoperfusion.^28^, ^29^ The no‐reflow phenomenon, defined in animal models as impaired microvascular perfusion despite successful macrovascular recanalization, is the most recognized subtype and has been extensively investigated for decades. First introduced by Ames et al in 1968, it was observed in rabbits in which prolonged global ischemia led to incomplete microvascular flow restoration, mainly due to compressive vasogenic edema and erythrocyte‐induced capillary obstruction.^30^, ^31^ Consistently, subsequent studies across various animal models of global or focal ischemia have confirmed the presence of microcirculatory disturbances and reperfusion deficits within infarcted regions following clip or thrombus removal.^32^, ^33^, ^34^ However, the concepts of “incomplete tissue‐level reperfusion” and “no‐reflow” have recently often been used interchangeably, which has introduced considerable conceptual ambiguity.

The other major mechanism underlying tissue‐level hypoperfusion despite recanalization is distal emboli, which predominantly manifest after incomplete (eTICI 2b–2c) recanalizations.^11^, ^35^ Fragmented thrombi occlude distal arterial branches, causing territorial hypoperfusion characterized by wedge‐shaped patterns within the initially affected vascular territory, which may progress to new infarcts. This type of hypoperfusion is most commonly observed after incomplete recanalization (eTICI 2b–2c) but can also occur in patients achieving complete angiographic recanalization (eTICI 3).^8^, ^11^, ^36^ These territorial hypoperfusions persist on 24 to 48‐hour follow‐up imaging in approximately 60% of these patients, leading to new infarcts beyond the initial core, whereas the rest experience spontaneous delayed reperfusion without tissue infarction.^13^, ^37^ Delayed reperfusion likely reflects spontaneous thrombolysis and recovery of microvascular function, with these biological processes demonstrating temporal patterns and anatomic correspondence between initially identified injury regions and subsequently reperfused territories, supporting genuine therapeutic recovery rather than initial assessment limitations.^38^ Detecting territorial hypoperfusion in eTICI 3 cases can prompt re‐examination of end‐procedure DSA, identifying missed distal occlusions and leading to eTICI downgrading.^11^ This form of incomplete tissue‐level reperfusion likely responds to adjunctive intra‐arterial thrombolysis, which can dissolve per‐procedural distal emboli and in turn improve reperfusion.

In contrast, in patients with core‐lab confirmed complete recanalization (eTICI 3), persistent hypoperfusion has been mainly reported to occur within established ischemic cores and is thought to result from microvascular dysfunction‐related no‐reflow. Based on preclinical studies, this type of incomplete tissue‐level reperfusion is due to entirely distinct underlying mechanisms, and likely requires alternative therapeutic strategies.^39^ Of note, no‐reflow is likely to also occur in eTICI < 3 grades within the ischemic core, in conjunction or not with territorial hypoperfusions, although this has not been the subject of specific reports as of today. In clinical practice, patients may exhibit both mechanisms simultaneously, with territorial hypoperfusion affecting previously uninfarcted regions due to distal emboli, whereas no‐reflow affects the established ischemic core due to microvascular dysfunction. This coexistence explains the heterogeneity in reported hypoperfusion rates and treatment responses, emphasizing the need for comprehensive perfusion assessment that can characterize both mechanisms.

It is important, however, to underscore that hypoperfusion on post‐thrombectomy imaging represents a common manifestation potentially arising from a diversity of underlying causes.^27^ In addition to distal emboli and microvascular no‐reflow, as highlighted above, various other factors may also contribute. Proximal vascular abnormalities, including residual stenosis, re‐occlusion, or underlying intracranial atherosclerotic disease at the site of the primary occlusion, may impair downstream flow.^40^, ^41^ Moreover, procedural complications, such as vessel dissection, vasospasm, or delayed re‐occlusion, may result in secondary perfusion deficits.^42^, ^43^ In some cases, mass effect from post‐procedural hemorrhagic transformation or malignant edema may compromise cerebral perfusion in adjacent territories.^44^, ^45^, ^46^, ^47^ Due to their well‐defined nature and established pathophysiological pathways, these causes of hypoperfusion will not be further elaborated upon in the present review, but must be excluded when assessing no‐reflow and territorial hypoperfusions in clinical studies.

## Assessment of Incomplete Tissue‐Level Reperfusion After Thrombectomy

Although substantial progress has been made in understanding the prevalence and patterns of incomplete tissue‐level reperfusion despite recanalization, critical questions remain regarding the optimal assessment methodologies (including timing and perfusion technique), precise classification, temporal evolution, differential impact on outcome, and therapeutic implications. To address these knowledge gaps, the present comprehensive review synthesizes findings from contemporary studies investigating incomplete tissue‐level reperfusion following successful recanalization (m/eTICI ≥ 2b).

### Temporal Considerations

As shown in Table 1, assessing incomplete tissue‐level reperfusion can yield distinct results according to the timepoint since EVT due to delayed spontaneous reperfusion, and potentially also delayed hypoperfusion.^13^, ^48^ Accordingly, the variability in reported hypoperfusion prevalence may partly depend on timing of assessment. As already pointed out, a recent systematic review and meta‐analysis of 6 studies reported that approximately 41% (interquartile range = 33%–51%) of patients with early territorial hypoperfusion experience delayed spontaneous reperfusion within 24 hours post‐intervention,^13^ complicating the accurate assessment of incomplete tissue‐level reperfusion and potentially confounding treatment efficacy assessment in trials. Thus, one study reported an 80% incidence of hypoperfusion (defined as time of maximum plasma concentration [Tmax] > 6 seconds) in patients with TICI 2b to 3 as assessed 30 minute post‐thrombectomy.^36^ However, perfusion assessments carried out approximately 24 hours post‐thrombectomy revealed spontaneous reperfusion rates of 30.7% to 61.5% in territorial regions corresponding to angiographically incomplete recanalization.^8^, ^35^, ^36^, ^38^, ^49^, ^50^, ^51^, ^52^, ^53^ Another study evaluating magnetic resonance perfusion at 48 hours post‐thrombectomy reported a spontaneous delayed reperfusion rate of 47.8%.^54^ Importantly, a recent study highlighted that delayed reperfusion may be defined not only as reperfusion of a previously hypoperfused area but also as the absence of hypoperfusion 24 hours post‐thrombectomy in TICI grades < 3, thus broadening the definition of this phenomenon.^35^ This concept therefore purports that patients graded as eTICI 2b to 2c with normal perfusion at 24 hours must have had initial hypoperfusion (given their incomplete DSA‐based recanalization) that subsequently reperfused spontaneously. Overall, therefore, hypoperfusion post‐EVT needs to be defined not just as territorial versus no‐reflow, but also according to the timing of the assessment.

**TABLE 1:** Literature Review of the Characteristics and Outcomes of Incomplete Tissue‐Level Reperfusion After Successful Thrombectomy^a^

Study	TICI grade^b^	Definition	Timing/method	Region	Exclusion criteria^c^	Hypoperfusion, *N* (%)^d^	Outcome impact
1a. Studies reporting no‐reflow with exclusion of confounding factors							
ter Schiphorst 2021^56^	mTICI≥ 2c	rCBF≥40% decrease	24hr/ ASL	Core/new infarct	A, B, C	2c: 1/11 (9.1%) 3: 1/11 (9.1%)	NA
Ng 2022^15^	eTICI ≥ 2c	rCBV/rCBF>15% decrease	24 ±6 hr/ CTP or MRP	Core	A, B, C^e^	2c: 15/73 (20.5%) 3: 18/57 (31.6%)	Yes
Luijten 2023^71^	eTICI ≥ 2b	CBF≥40% decrease	24 hr/ MRP	Core	A, B, C	2c: 0/9 (0%) 3: 0/18 (0%)	NA
Mujanovic 2024 (a)^69^	eTICI 3	Visual assessment	24hr/ MRI and CTP	Core	A, B, C	2c: NR 3: 14/248 (5.6%)	Yes
Mutimer 2024^70^	eTICI ≥ 2c	CBV/CBF>15% asymmetry	24±6hr/ CTP or MRP	Core	A, B, C	2c: 15/69 (22%) 3: 14/62 (23%)	Yes
	eTICI ≥ 2c	>40% CBF asymmetry	24±6hr/ CTP or MRP	Core	A, B, C	2c: 1/69 (1%) 3: 0/62 (0%)	NA
Rivet 2025^14^	eTICI ≥ 2c	rCBV/rCBF>15% asymmetry	24±6 hr / CTP or MRP	Core	A, B, C	2c‐3: 30/150 (20%)	Yes
Valls Carbó 2025^72^	mTICI ≥2C	>15% asymmetry	2hr/PWI	Core	A, B, C	2c: 4/11 (36.4%) 3: 4/22 (18.2%)	Yes
Hernandez Petzsche 2025^67^	eTICI ≥ 2b	>15% asymmetry	Median 4 days / ASL	Core	C	2c‐3: 10/86 (11.6%)	Yes
1b. Studies reporting no‐reflow without exclusion of confounding factors							
Potreck 2021^45^	mTICI≥ 2b	Visual assessment	24hr/ MRP	Core	NR	2c: 3/13 (23.1%) 3: 2/16 (12.5%)	NA
1c. Studies that did not distinguish between no‐reflow and territorial hypoperfusion							
Rubiera 2020^55^	mTICI 2a‐3	Tmax > 6s	Within 30 min/CTP	Total territory	NR	2c: NR 3: 40/94 (42.5%)	Yes
Tan 2021^49^	mTICI ≥ 2b	<90% reduction of pre‐MT Tmax >6s lesion volume	24‐36hr/CTP or MRP	Total territory	NR	2c: 3/15 (20%) 3: 3/16 (18.8%)	NR
Laredo 2022^54^	eTICI ≥ 2b	Tmax > 6s	48hr/ MRP	Total territory	NR	2c: 3/8 (37.5%) 3: 3/13 (23.1%)	Yes
Luby 2022^50^	mTICI 0‐3	Tmax>6s and volume>10 mL	24hr/ MRP	Total territory	B	2c‐3: 2/27 (7%)	NA
Mujanovic 2022^8^	eTICI ≥ 2a	Tmax≥4s	24 ±12 hr/ MRP and CTP	Total territory	B, C	2c: 25/144 (17%) 3: 0/194 (0%)	Yes in eTICI2c
Bai 2023^53^	mTICI≥ 2b	<90% reduction of pre‐MT Tmax>6s volume	Within 72h / CTP	Total territory	B	2c‐3: 10/44 (23%)	Negative impact
Hong 2023^81^	mTICI≥2a	DT> 3s, RI < 0.9	24‐48hr/CTP	Total territory	NR	2c: 2/6 (33.3%) 3: 10/30 (33.3%)	NR
Mujanovic 2024 (b)^68^	eTICI ≥ 2a	Focal, wedge‐shaped perfusion delay	24±12hr/ CTP	Total territory	NR	NR	NA
Mujanovic 2025^35^	eTICI 2a‐2c	Focal, wedge‐shaped perfusion delay	24±12 hr / CTP or MRP	Total territory	NR	NR	NA

^a^ Studies of anterior circulation LVO achieving successful recanalization, reporting no‐reflow incidence (m/eTICI 2c‐3) or delayed/persistent territorial hypoperfusion.

^b^ All studies used independent central core laboratory assessment of TICI grades, except: Ng 2022 (assessment method not specified), Hernandez Petzsche 2025, Potreck 2021, Rubiera 2020, Luby 2022, Bai 2023, and Mujanovic 2025 (mixed: EXTEND‐IA trials used central core lab assessment, other studies varied).

^c^ Exclusion criteria include A, ICA stenosis/occlusion; B, intracranial re‐occlusion; C, hemorrhagic transformation.

^d^ Delayed reperfusion rates: Among studies assessing territorial hypoperfusion, delayed reperfusion (defined as absence of new perfusion deficits/infarcts in initially occluded territory on typically 24h follow‐up despite eTICI<3) occurred in: Tan 2021: 24/59 (40.7%, eTICI 2b‐2c); Laredo 2022: 11/23 (47.8%, eTICI 2b‐2c); Luby 2022: 8/23 (34.7%, mTICI 0‐2c); Mujanovic 2022: 228/372 (61.3%, eTICI 2a‐2c); Bai 2023: 10/19 (52.6%, mTICI 2b); Mujanovic 2024(b): 108/187 (57.8%, eTICI 2b‐2c); Mujanovic 2025: 512/832 (61.5%, eTICI 2a‐2c).

^e^ Ng 2022 did not exclude patients with hemorrhagic transformation but excluded hemorrhagic zones from ROIs during automated perfusion analysis.

ASL = arterial spin labeling; CBF = cerebral blood flow; CBV = cerebral blood volume; CTP = computed tomography perfusion; DT = delay time; ICA = internal carotid artery; LVO = large vessel occlusion; m/eTICI = modified/extended thrombolysis in cerebral infarction; MRP = magnetic resonance perfusion; NA = not assessable; NR = not reported; PWI = perfusion‐weighted imaging; rCBF = relative CBF; rCBV = relative CBV; RI = reperfusion index; Tmax = time‐to‐maximum.

The timing of perfusion assessment also introduces important clinical implications. Thus, immediate post‐thrombectomy imaging is relevant not just mechanistically but also clinically, as it enables the identification of hypoperfused regions that may still be amenable to rescue therapies before irreversible tissue injury occurs. In contrast, as already alluded, assessments performed at 48 hours may entirely miss perfusion deficits that were present earlier but have spontaneously resolved, potentially reflecting so‐called “non‐nutritional” reperfusion that occurred too late to salvage brain tissue. As emphasized in recent studies,^11^, ^36^, ^55^ early detection of residual perfusion deficits is crucial for identifying patients who may benefit from adjunctive interventions.

Regarding future studies, immediate post‐EVT imaging seems to be the best timing to capture both no‐reflow and territorial hypoperfusions; 24‐hour imaging with standardizing delayed reperfusion criteria is pivotal for prognostication and as trial surrogate end point; whereas later imaging may help refine the understanding of irreversible versus reversible microvascular injury. Obviously, serial perfusion imaging at both the ultra‐early and the 24‐hour timepoints appears particularly relevant to decipher the mechanisms as well as evaluate the evolution of hypoperfusions, natural or post‐treatment.

### Imaging Modalities and Techniques

Several previous reviews have explored the methods of evaluation of hypoperfusion following thrombectomy.^17^, ^25^, ^26^ However, a consensus standardized definition of post‐EVT incomplete tissue‐level perfusion is still lacking.^56^ Perfusion imaging techniques, such as CT perfusion (CTP), perfusion‐weighted imaging (PWI), and (in early studies) single‐photon emission computed tomography (SPECT), have been widely used in clinical practice to assess cerebral reperfusion status.^57^ These modalities, however, exhibit significant variability due to differences in evaluation variable (eg, CBF, cerebral blood volume [CBV], and Tmax), definition of hypoperfusion (eg, extent, within or outside the initial core), and cutoff values.^27^ Establishing standardized thresholds for incomplete tissue reperfusion remains challenging due to variations in imaging protocols and variables, software vendors, patient factors, and institutional differences.^58^ Whereas landmark trials used specific thresholds (Tmax > 6 seconds for penumbra, and CBF < 30% for core),^59^, ^60^ these are context‐specific and may require adjustment based on arterial input function selection and stroke subtypes. Ideal standardization should ensure that patients with comparable post‐thrombectomy hypoperfusion and infarct volumes receive consistent diagnoses, regardless of imaging modality or post‐processing software. Notably, Ng et al demonstrated that a > 15% asymmetry in CBV or CBF in the ischemic region was strongly associated with functional outcomes, although this relatively low threshold might potentially capture other phenomena beyond no‐reflow.^15^ Other studies have proposed a stricter ≥ 40% reduction in CBF relative to the contralateral hemisphere as threshold for defining no‐reflow.^56^ Although Tmax, which is heavily weighted toward macrovascular tracer transit,^61^ has been utilized according to a > 4‐second or > 6‐second threshold to identify territorial hypoperfusions related to distal emboli,^11^ its interpretation requires caution as this timing parameter can be confounded by collateral circulation, arterial input function selection, and various non‐pathological factors, limiting its direct relationship to underlying stroke physiology.

Several challenges limit the accurate assessment of post‐EVT incomplete tissue‐level reperfusion. One such challenge lies in the accurate delineation of the ischemic core itself, as commonly used thresholds such as relative cerebral blood flow (rCBF) < 30% may underestimate or overestimate the true extent of irreversible tissue damage.^62^, ^63^ Whereas diffusion‐weighted imaging provides greater accuracy, particularly when using an ADC threshold, such as 620 × 10^−6^ mm^2^/s, it remains imperfect in defining core boundaries.^64^ Moreover, existing perfusion assessment techniques are limited by their costs, restricted accessibility for critically ill patients, and exposure to ionizing radiation and potential renal toxicity associated with CT, compromising their repeatability and dynamic monitoring capabilities.^65^ Therefore, standardized, accessible, and reproducible imaging methods are critically needed for accurate assessment of post‐EVT incomplete tissue‐level reperfusion. CTP and perfusion magnetic resonance imaging (MRI) have nominal voxel sizes of 1 to 2 millimeters, but their actual ability to detect focal perfusion deficits is limited by signal‐to‐noise ratio and partial volume effects, making detection of patchy microvascular hypoperfusion challenging. Arterial spin labeling (ASL), a quantitative MRI‐based technique that does not require external contrast agent, is particularly attractive as it uses a freely diffusible tracer (namely, incoming arterial blood water) allowing to map true capillary perfusion, and produces quantitative CBF parametric maps.^66^, ^67^ Further research is therefore necessary to establish standardized definitions, multi‐parametric approaches, and optimal cutoff values in the evaluation of post‐EVT hypoperfusion.

### Refining the Angiographic Assessment and Grading of Post‐Thrombectomy Success

The traditional reperfusion grading tools such as the e/mTICI scale are widely used for evaluating macrovascular recanalization but may not accurately reflect microvascular perfusion status. Moreover, studies have demonstrated significant interobserver variability in mTICI scoring, with discrepancies observed in up to 25% of cases, including a tendency for operators to overestimate reperfusion grades compared to core laboratory assessment, potentially leading to misclassification of reperfusion success.^11^, ^68^ This variability is partly due to the scale's broad categories, which may lack the granularity needed to characterize the extent of reperfusion precisely. According to a new angiographic reclassification framework, the detection of territorial hypoperfusion (via cone‐beam CT or dynamic angiography) warrants downgrading eTICI 2c to eTICI 2b, thereby refining prognostication and identifying potential thrombolysis‐responsive subgroups.^11^, ^68^ This dynamic temporal evolution has significant implications for treatment selection, emphasizing the need for standardized assessment timepoints in neuroimaging protocols to classify reperfusion status and guide therapeutic decision making. Based on the above recent reports, one may consider incorporating the assessment of immediate post‐EVT perfusion status into a modified TICI scale.

## The Two Main Subtypes of Incomplete Tissue‐Level Reperfusion

### No‐Reflow Despite Successful Recanalization

The reported prevalence of no‐reflow varies considerably depending on operational definition, study population, and assessment techniques.^27^ A recent systematic review, focusing solely on patients achieving complete reperfusion (m/eTICI 3) and accounting for straightforward confounding factors (parenchymal hemorrhage, proximal re‐occlusion, and carotid occlusion), reported a no‐reflow incidence ranging from 0% (0/194 patients) to 9.1% (4/33 patients), significantly lower than previously reported rates.^56^, ^69^, ^70^, ^71^ When including patients with near‐complete reperfusion (eTICI 2c) or those with hemorrhagic transformation, the apparent prevalence of hypoperfusion within the ischemic core increased considerably. One study reported a prevalence of no‐reflow approximately 20% in eTICI 2c and 31% in eTICI 3, which could be overestimated for methodological reasons.^15^ Conversely, another study, by excluding the above 3 confounders, reported a low no‐reflow prevalence of 6% in patients with eTICI 3, consistent with the above findings.^69^ However, a more recent article, also excluding these major confounders, reported a higher, though still modest, no‐reflow prevalence of 18% in patients with eTICI 3.^72^ Overall, the reported findings so far suggest a no‐reflow prevalence probably approximately 10% in the eTICI 3 grade. However, the exact prevalence of no‐reflow will remain uncertain pending formal validation of perfusion thresholds by means of experimental imaging‐histopathological correlation studies, preferably in nonhuman primates.^27^

The choice of quantitative thresholds likely significantly impacts the reported incidence: a > 15% asymmetry in CBV or CBF identified hypoperfusion in 10.7% of patients with eTICI 2c and 11.5% of patients with eTICI 3,^70^ whereas stricter criteria (> 40% CBF asymmetry) yielded substantially lower rates.^69^, ^70^ Emerging evidence suggests that “no‐reflow” impacts functional outcomes, although the clinical significance of true microvascular dysfunction remains less well characterized compared to territorial hypoperfusion and likely requires alternative therapeutic strategies targeting microvascular pathophysiology rather than thrombolytic approaches. For rigorous assessment, the true no‐reflow's impact should ideally be evaluated only in patients with confirmed complete recanalization (eTICI 3), free from confounding factors, such as parenchymal hemorrhage, re‐occlusion, per‐procedural complications, or proximal cervical occlusion. However, such comprehensive data are currently limited in the literature. Even less known is the prevalence of no‐reflow in eTICI2b‐3 grades. Thus, clinical research on no‐reflow currently aims to determine its prevalence, spatial extent and clinical implications, with the ultimate goal of designing appropriate trials of therapeutic agents targeting no‐reflow, using perfusion imaging to monitor treatment effects.

### Territorial Hypoperfusion

Territorial hypoperfusion represents the most prevalent subtype of incomplete tissue‐level reperfusion, although its rates vary dramatically according to eTICI grade. Flat‐panel CT perfusion imaging performed immediately after thrombectomy reveals these patterns in approximately 90% of patients with eTICI 2b, and, as expected, far less frequently in patients with eTICI 2c (approximately 17%) and even less in patients with eTICI 3 (approximately 11%).^8^, ^11^, ^36^ A defining characteristic is its dynamic temporal evolution, with approximately 40% of patients experiencing spontaneous delayed reperfusion within 24 hours, likely reflecting spontaneous thrombolysis of residual distal emboli.^13^, ^37^ Patients with persistent territorial hypoperfusion at 24 hours demonstrate significantly poorer functional outcomes compared with those achieving delayed reperfusion, with the persistent pattern leading to new infarcts beyond the initial core.^8^, ^13^, ^37^ Unlike microvascular dysfunction, territorial hypoperfusion may be responsive to adjunctive intra‐arterial thrombolysis, with recent RCTs suggesting greater benefit in patients with lower reperfusion grades (eTICI 2b) who predominantly exhibit this pattern, although these findings require careful interpretation given cross‐trial variability.^73^, ^74^, ^75^, ^76^, ^77^ For optimal therapeutic decision making, territorial hypoperfusion should be assessed through immediate post‐procedural perfusion imaging to identify patients who may benefit from adjunctive interventions before irreversible tissue injury occurs.

### Differentiating Territorial Hypoperfusion from No‐Reflow

**FIGURE 2:**
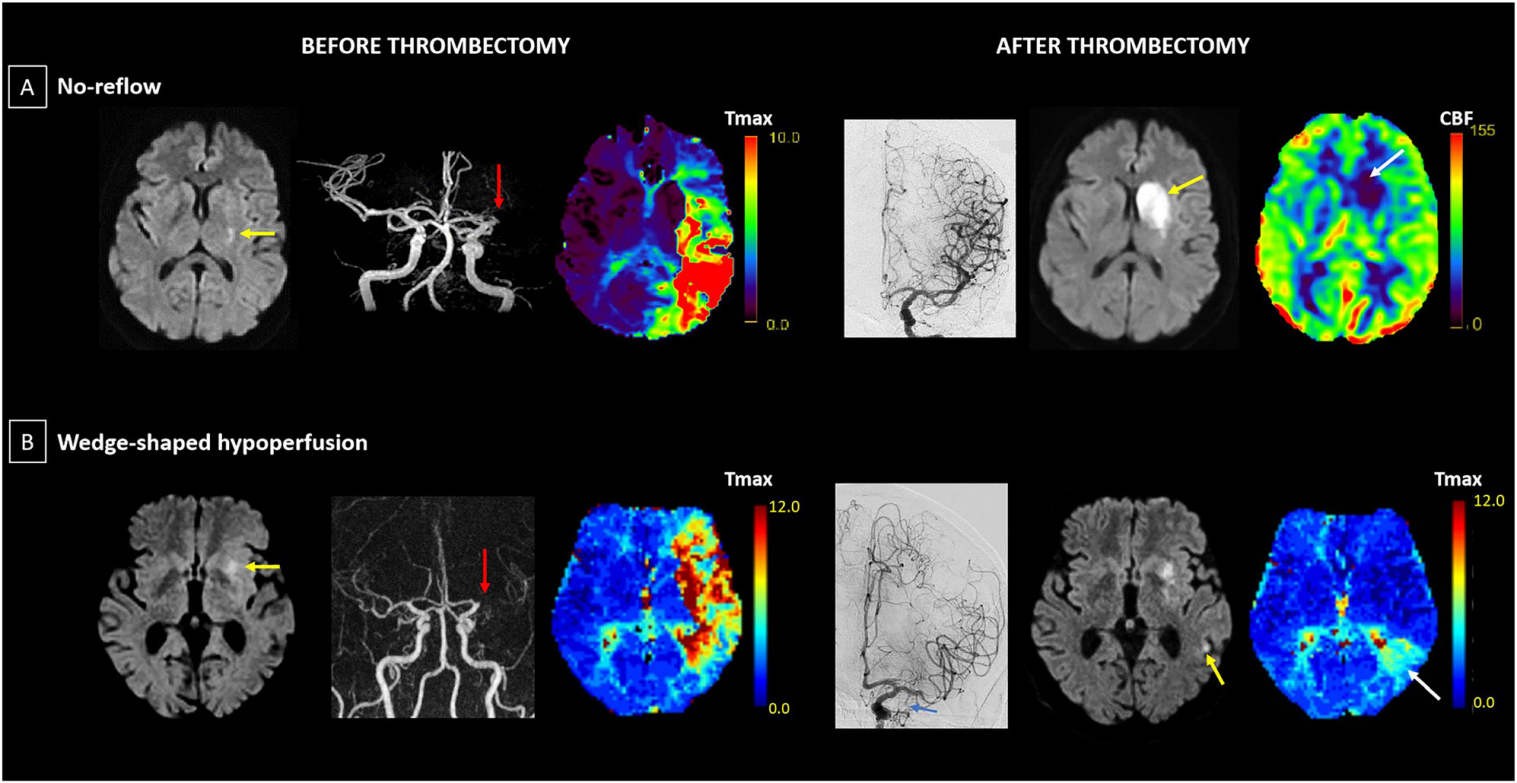
No‐reflow versus wedge‐shaped hypoperfusion after successful thrombectomy. Panel (A) “No‐reflow” pattern (Patient 1; adapted from [ter Schiphorst et al]^56^). The patient was admitted for hemiparesis and global aphasia with partial improvement on arrival resulting in an NIHSS of 4. Left‐to‐right, images acquired before thrombectomy (MRI, obtained 84 minutes after symptom onset): (1) DWI showing the baseline ischemic core (*yellow arrow*); (2) time‐of‐flight angiography demonstrating an M1 occlusion (*red arrow*); and (3) Tmax perfusion maps (in seconds) with a large delay in the corresponding territory. The patient was treated by thrombectomy 160 minutes after symptom onset. Left‐to‐right, images acquired after thrombectomy: (4) final DSA documenting successful recanalization (mTICI 3); (5) DWI at approximately 24 hours showing infarct extension to the entire caudate and lentiform nuclei (*yellow arrow*); and (6) ASL‐CBF (in ml/100 g/min) at approximately 24 hours revealing “no‐reflow” in the newly infarcted territory (*white arrow*). There was no evidence of re‐occlusion or hemorrhagic transformation on the approximately 24 hour MRI (MRA and T2*; not shown). Panel (B) “Wedge‐shaped hypoperfusion” pattern (Patient 2; in‐house case). The patient was admitted for facial paralysis and dysarthria, with an initial NIHSS score of 2. Left‐to‐right, images acquired before thrombectomy (MRI, obtained 95 minutes after symptom onset): (1) DWI showing the baseline ischemic core (*yellow arrow*); (2) contrast‐enhanced MRA (gadolinium) confirming an M1 occlusion (*red arrow*); and (3) Tmax perfusion maps (in seconds) with a territory‐concordant perfusion delay. The patient was initially treated with intravenous thrombolysis only, administered 120 minutes after symptom onset. Due to neurological deterioration leading to an NIHSS score of 6, a thrombectomy was subsequently performed 300 minutes after symptom onset. Left‐to‐right, images acquired after thrombectomy: (4) final DSA documenting an mTICI2b recanalization with persistence of an inferior branch occlusion (*blue arrow*); (5) DWI at approximately 24 hours showing an infarct growth (*yellow arrow*); (6) Tmax perfusion maps (in seconds) at approximately 24 hours showing a wedge‐shaped hypoperfusion in the same territory (*white arrow*), consistent with a distal embolus, which was visible in the concomitant time‐of‐flight imaging (not shown). ASL = arterial spin labeling; CBF = cerebral blood flow; DWI = diffusion‐weighted imaging; DSA = digital subtraction angiography; M1 = first segment of the middle cerebral artery; MRA = magnetic resonance angiography; MRI = magnetic resonance imaging; mTICI = modified treatment in cerebral infarction; NIHSS = National Institutes of Health Stroke Scale; Tmax = time to maximum concentration. [Color figure can be viewed at www.annalsofneurology.org]

In clinical practice, distinguishing no‐reflow from distal per‐procedural embolization requires careful analysis of perfusion imaging patterns, timing, and angiographic correlation. Figure 2 illustrates these contrasting patterns in 2 patients with similar pre‐thrombectomy presentation (left MCA occlusion). Despite both achieving successful recanalization, case A demonstrates no‐reflow with persistent hypoperfusion confined within the expanded ischemic core despite mTICI 3 recanalization (see Fig 2A), whereas case B shows the characteristic wedge‐shaped territorial hypoperfusion pattern extending to cortical regions corresponding to residual distal vessel involvement (see Fig 2B). These distinct imaging signatures highlight the importance of comprehensive perfusion assessment in identifying the predominant mechanism of incomplete tissue‐level reperfusion. Territorial hypoperfusion typically manifests as wedge‐shaped deficits within or at the borders of the initially affected vascular territory, defined using Tmax maps.^11^, ^54^ Thus, for optimal detection, territorial hypoperfusions should be assessed as early as possible, and, if possible, immediately after the end of the endovascular procedures.

Conversely, no‐reflow is thought to represent microvascular perfusion failure within the initial or 24‐hour ischemic core region despite successful macrovascular recanalization, potentially affecting tissue that might otherwise escape pan‐necrosis.^34^, ^78^, ^79^, ^80^ Although this phenomenon is observed immediately after reperfusion in animal models, its precise characteristics and temporal evolution in humans remain incompletely understood.^25^ Clinically, no‐reflow has been typically assessed using CBF or CBV derived from perfusion imaging modalities (such as MRI‐based PWI or ASL, or CT perfusion) at 24 to 48 hours post‐thrombectomy, which reflects final infarct extent and microcirculatory perfusion status.^56^, ^81^ In addition, delayed hypoperfusion, which in animal models may develop secondarily within necrotic tissue, may be confused for no‐reflow on 24 to 48‐hour perfusion imaging.^48^ Accordingly, early perfusion assessments are also preferable when studying no‐reflow.

## Therapeutic Strategies for Territorial Hypoperfusion and No‐Reflow

### Intra‐Arterial Thrombolysis to Address Territorial Hypoperfusion

Recent RCTs have provided breakthrough evidence regarding the efficacy of intra‐arterial thrombolysis following successful mechanical thrombectomy to address incomplete tissue‐level reperfusion (Table 2).^73^, ^74^, ^75^, ^76^, ^77^, ^82^, ^83^ Although the POST‐TNK and POST‐UK trials, which recruited patients with higher reperfusion grades (eTICI ≥ 2c), failed to demonstrate significant clinical benefit,^74^, ^75^ the CHOICE, ANGEL‐TNK, and PEARL trials, which included patients with eTICI ≥ 2b50, generally showed positive outcomes.^73^, ^76^, ^77^ Recent meta‐analysis evidence suggested that the benefit of intra‐arterial thrombolysis varies across recanalization grades,^84^ with eTICI 2b reperfusion appearing to derive greater benefit from intra‐arterial thrombolysis compared with those achieving more complete reperfusion (eTICI 2c/3).^73^, ^74^, ^75^, ^76^, ^77^ However, these observations are influenced by cross‐trial comparisons, differences in thrombolytic agents, study populations, and other design factors, and thus should be interpreted with caution.

**TABLE 2:** Impact of Intra‐Arterial Thrombolysis After Thrombectomy on Reperfusion and Clinical Outcomes in Randomized Controlled Trials^a^

Study	Patients	IVT before EVT	IA dose	eTICI grade^b^	Primary outcome^c^ (mRS 0‐1, 90d)	Treatment effect by eTICI subgroup^d^
CHOICE 2022^76^	AC/PC, N=113	ALT: n=38 vs PLA: n=31	ALT 0.225 mg/kg (max 22.5 mg)	≥2b50	ALT 59% vs PLA 40.4% (Sig)	2b: NS ≥2c: Sig
Laredo et al 2022^54^ (sub‐study of CHOICE)^e^	AC/PC, N=36	ALT: n=7 vs PLA: n=7	ALT 0.225 mg/kg (max 22.5 mg)	≥2b50	Abnormal perfusion: ALT 24% vs PLA 58% (Sig)	NR
ATTENTION‐IA 2024^82^	PC, N=208	TNK: n=28 vs Control: n=25	TNK 0.0625 mg/kg (max 6.25 mg)	≥2b50	TNK 34.6% vs Control 26%	2b: NS ≥2c: NS
POST‐UK 2025^75^	AC (ICA/M1/M2), N=532	None	UK 100,000 IU	≥2c	UK 45.1% vs Control 40.2%	2c: NS 3: NS
POST‐TNK 2025^74^	AC (ICA/M1/M2), N=539	None	TNK 0.0625 mg/kg (max 6.25 mg)	≥2c	TNK 49.1% vs Control 44.1%	2c: NS 3: NS
ANGEL‐TNK 2025^77^ ^,^ ^e^	AC (ICA/M1/M2), N=255	None	TNK 0.125 mg/kg (max 12.5 mg)	≥2b50	TNK 40.5% vs Control 26.4% (Sig)	2b: Sig ≥2c: NS
PEARL 2025^73^	AC (ICA/M1/M2), N=324	ALT: n=69 vs Control: n=66	ALT 0.225 mg/kg (max 20 mg)	≥2b50	ALT 44.8% vs Control 30.2% (Sig)	2b: NS ≥2c: NS
DATE 2025^83^	AC (ICA/M1/M2), N=157	None	TNK 0.03125mg/kg	≥2b50	TNK 37.0% vs Control 33.8%	NR
			TNK 0.0625mg/kg		TNK 43.5% vs Control 33.8%	

^a^ All trials were randomized controlled trials comparing intra‐arterial thrombolysis versus placebo or no treatment after mechanical thrombectomy in patients with large vessel occlusion acute ischemic stroke.

^b^ Independent central core laboratory assessment was performed in CHOICE, Laredo et al (substudy of CHOICE), POST‐UK, POST‐TNK, and PEARL. The remaining studies did not use independent central assessment.

^c^ Primary outcome significance: Statistically significant differences (*P* < 0.05 or marked as ‘Sig’ in table) were observed in: CHOICE (*P* = 0.047), Laredo et al substudy (*P* = 0.03 for abnormal perfusion at 48h), ANGEL‐TNK (*P* = 0.02), and PEARL (*P* = 0.01). Non‐significant results (NS) were observed in: ATTENTION‐IA (*P* = 0.12), POST‐UK (*P* = 0.19), POST‐TNK (*P* = 0.11), and DATE (*P* = 0.5 for both doses).

^d^ Treatment Effect by eTICI Subgroup: NS, not significant; Sig., statistically significant (*P* < 0.05 or 95% CI excludes null value).

^e^ Post‐thrombectomy perfusion imaging was performed in only two trials. Laredo et al 2022 (sub‐study of CHOICE) performed MRP at 48 h post‐EVT, and ANGEL‐TNK 2025 performed CTP at 24±12 h post‐mechanical thrombectomy. All other trials (CHOICE 2022, ATTENTION‐IA 2024, POST‐UK 2025, POST‐TNK 2025, PEARL 2025, and DATE 2025) did not perform systematic post‐procedural perfusion imaging.

Abbreviations: AC = anterior circulation; aRR = adjusted risk ratio; ALT = alteplase; CTP = Computed Tomography Perfusion; eTICI = expanded thrombolysis in cerebral infarction; EVT = Endovascular Thrombectomy; IA = intra‐arterial; ICA = internal carotid artery; IVT = intravenous thrombolysis; M1/M2 = MCA segments; MCA = Middle Cerebral Artery; MRP = Magnetic Resonance Perfusion; mRS = modified Rankin Scale; MT = mechanical thrombectomy; NR = not reported; PC = posterior circulation; PLA = placebo; TNK = tenecteplase; UK = urokinase.

Consistent with this observation, and as discussed above, the vast majority of patients with eTICI 2b have distal emboli that likely respond to thrombolytic dissolution, whereas a fraction of patients with higher reperfusion grades and persistent hypoperfusion experience no‐reflow, which may likely be less responsive to thrombolysis and might even be associated with a higher risk of hemorrhagic transformation due to blood–brain barrier disturbance. This hypothesis suggests that the optimal candidates for post‐thrombectomy thrombolytic therapy are those with territorial hypoperfusion from distal emboli rather than those with isolated no‐reflow. Furthermore, when incomplete tissue‐level reperfusion results from mechanical obstruction by distal thrombi, the composition of the latter likely entails significant heterogeneity and evolves over time, influencing their susceptibility to thrombolytic therapy. Histological studies of proximal thrombi extracted during EVT have identified 2 distinct types: red blood cell‐rich thrombi, composed primarily of packed red blood cells entangled within a loose fibrin meshwork^85^; and platelet‐rich thrombi, characterized by the presence of platelets embedded within a dense fibrin matrix, alongside significant amounts of von Willebrand factor and extracellular DNA,^86^ which are known to resist thrombolytics.^87^

### Preclinical Research Targets for True “No‐Reflow”

Whereas it is well‐established that no‐reflow arises from microvascular/capillary abnormalities, the precise underlying mechanisms remain elusive, with multiple factors appearing to contribute (Supplementary Table S1).

Although no‐reflow is recognized after vascular recanalization, it actually develops during the ischemic phase, and its intensity is directly linked to the duration of occlusion.^88^ Previous middle cerebral artery (MCA) occlusion studies in nonhuman primates demonstrated that no‐reflow primarily affects the deep MCA territory and worsens with extended occlusion duration, whereas comparable degrees of no‐reflow were observed in both temporary and permanent occlusions of equal duration, as well as with variable durations of reperfusion for a given occlusion time, providing strong evidence for an intra‐ischemic phenomenon.^34^, ^89^, ^90^, ^91^ Accordingly, neuronal death appears to precede microvascular blockade and to be more extensive than the area of perfusion abnormality.^34^ Furthermore, mechanisms involved in no‐reflow, such as pericyte contraction,^39^ endothelial damage,^33^, ^92^ and neutrophil infiltration, begin during arterial occlusion and persist after vessel recanalization.^88^, ^93^, ^94^ Based on this evidence, treating no‐reflow as early as possible during the occlusion phase would appear more reasonable than after recanalization, when microcirculation may be more difficult to reverse due to established cellular and molecular changes.

An important mechanism underlying capillary flow stagnation involves polymorphonuclear leukocyte plugging within the lumen.^33^ As demonstrated in preclinical models, targeting leukocyte‐endothelial interactions using anti‐E‐selectin and anti‐Ly6G antibodies can partially prevent neutrophil adhesion and improve CBF with studies showing up to 2.6‐fold increase in flow and significant reduction in infarct volume.^80^, ^95^ In addition, preclinical studies show that pericyte contraction can be reversed through several approaches, all demonstrating significant improvements in tissue perfusion in rodent models.^96^, ^97^ Regarding endothelial and astrocytic edema that narrow the lumen, interventions including squalenyl adenosine nanoparticles and regional hypothermia targeting AQP4 water channels have shown promise.^98^, ^99^ Additionally, several animal studies show nitric oxide (NO) donors can ameliorate no‐reflow by promoting vasodilation and improving microvascular patency after ischemia–reperfusion injury.^100^, ^101^, ^102^ Although these preclinical findings offer a wide array of potential interventions to prevent or treat no‐reflow in the clinical setting, none has yet been tested in humans. The clinical challenge lies in distinguishing which specific mechanisms underlie no‐reflow in individual patients, as current perfusion imaging cannot differentiate among pericyte constriction, neutrophil plugging, endothelial swelling, or other contributing factors. The application of one drug for all patients is unlikely to be universally effective. Importantly, identifying no‐reflow within established ischemic regions retains clinical relevance despite concurrent tissue damage. In the acute phase, detection of no‐reflow enables prognostic stratification to identify patients at highest risk of poor outcomes, advances mechanistic understanding of microvascular dysfunction, and helps distinguish it from territorial hypoperfusion due to distal emboli. Future work should prioritize multimodal approaches, including molecular imaging and biomarker strategies, capable of delineating the predominant mechanisms in individual patients, thereby enabling early, prevention‐oriented interventions administered at the pre‐EVT stage, before microvascular failure becomes irreversible and results in more extensive and homogeneously necrotic lesions.

### Limitations of Preclinical Research and Therapeutic Targets for No‐Reflow

Although laboratory models have advanced our understanding of no‐reflow, questions remain about their clinical translation. Animal studies often utilize direct in vivo visualization techniques like 2‐photon microscopy and laser Doppler imaging, or postmortem sectioning that cannot be replicated in patients with stroke.^103^, ^104^, ^105^, ^106^ This creates challenges in validating therapeutic targets and monitoring future treatment effects in clinical trials. Additionally, most experimental approaches use standardized occlusion methods (eg, thrombogenic coils or filaments) to induce controlled ischemia–reperfusion injuries, but these may not fully reproduce the complex pathophysiological mechanisms underlying no‐reflow that occur after complete recanalization in thrombectomy‐treated patients with stroke.^107^, ^108^ Multiple clinical factors, including duration of occlusion, collateral status, and various cellular events triggered by both ischemia and reperfusion, are difficult to simulate comprehensively in current models. Regarding clinical translation, the only currently proposed approach to identify no‐reflow after EVT is perfusion imaging, yet there has not been any validation of perfusion imaging as a surrogate of no‐reflow in animal models.

As pointed out earlier, some recent findings suggest that the presence of no‐reflow after successful thrombectomy markedly impacts clinical outcomes.^14^ This, in turn, suggests that current therapeutic approaches may be missing a critical therapeutic window. A recently published nonhuman primate study combining positron emission tomography (PET) and MRI reported a high incidence of apparent no‐reflow after recanalization, which predicted poor outcome.^79^ One significant caveat, however, is that the MCA occlusion technique used (coils) was unusual and may have prevented complete recanalization. Furthermore, postmortem data were not reported to confirm the presence of no‐reflow. This study, nevertheless, illustrates future directions for multi‐modality quantitative studies in nonhuman primates that should allow not only formal validation of perfusion imaging, including optimal variable and cutoff, to detect within‐core no‐reflow in the clinical setting, but also the testing of potentially clinically relevant interventions to prevent or treat no‐reflow. There is clinical evidence suggesting that certain patient subpopulations may be more susceptible to the no‐reflow phenomenon. These include patients with pre‐existing pro‐inflammatory conditions (such as diabetes and hyperlipidemia), structural brain abnormalities (such as white matter hyperintensities and pre‐stroke atrophy), elevated stroke‐induced inflammatory markers (such as interleukins and neutrophils), and those experiencing prolonged ischemic duration.^109^, ^110^, ^111^, ^112^ However, there are currently no systematic prospective studies to validate these risk factors and some studies have focused on populations with poor post‐thrombectomy outcomes rather than those with no‐reflow.

Altogether, the limitations of current evaluation methods and therapeutic strategies highlight the need for future research aimed at refining both the classification and targeted management of incomplete post‐EVT tissue‐level reperfusion, especially no‐reflow. Recommendations for future studies investigating incomplete tissue‐level reperfusion after successful recanalization are summarized in Table 3.

**TABLE 3:** Recommendations for Future Studies Investigating Incomplete Tissue‐Level Reperfusion After Successful Recanalization

	Recommendations
Study design	Prospective studies with serial perfusion imaging preferred over retrospective analyses. Independent core laboratory assessment using new‐m/eTICI classification. For territorial hypoperfusion: include patients with new‐m/eTICI 2b–2c. For no‐reflow: include only patients with new‐m/eTICI 3.
Confounding factors	Intracerebral hemorrhage, including parenchymal hematoma or confluent intracerebral hemorrhage. Hemodynamic‐degree upstream or proximal arterial stenosis not treated during the endovascular procedure. Re‐occlusion on follow‐up imaging. Record number of thrombectomy passes and use of thrombolytics/antithrombotics.
Assessment	***Timing*:** Immediate post‐EVT (< 30 min) plus 24 h follow‐up for territorial hypoperfusion; serial imaging (immediate +24 h), but no later than 72 h post‐EVT for no reflow assessment. ***Perfusion parameter*:** Tmax maps (sensitive to macrovascular occlusion) for territorial hypoperfusion; CBF, or CBV (sensitive to microvascular dysfunction) for no reflow. ***Hypoperfusion threshold*:** Use validated thresholds (eg, Tmax > 6 s for significant ischemia). ***Imaging pattern*:** Wedge‐shaped, territorial distribution for territorial hypoperfusion; patchy or uniform hypoperfusion within established infarct core for no reflow. ***Brain zones assessed*:** Initially affected vascular territory for territorial hypoperfusion; only pre‐thrombectomy ischemic core and surrounding areas for no reflow.
Therapies	For territorial hypoperfusion: Intra‐arterial thrombolysis or mechanical retrieval for distal emboli immediately post‐EVT if clinically indicated. For no reflow: Preventive neuroprotective strategies during ischemic phase (investigational).

CBF = cerebral blood flow; CBV = cerebral blood volume; eTICI = extended thrombolysis in cerebral infarction; EVT = endovascular thrombectomy; mTICI = modified thrombolysis in cerebral infarction; Tmax = time to maximum of the residue function.

## Conclusions

In summary, the persistent gap between large‐vessel recanalization and complete tissue reperfusion in AIS highlights the need for systematic, preferably sequential, brain perfusion assessment in prospective studies, moving beyond conventional angiographic grading. Despite achieving eTICI ≥ 2b, a significant proportion of patients experience poor outcomes, particularly due to persistent hypoperfusion, which underscores the limitations of current “successful” recanalization definitions. Implementing rapid perfusion imaging techniques, such as cone‐beam CT, upon completion of interventional procedures appears essential for the timely detection of perfusion deficits before irreversible injury develops. Emerging critical evidence distinguishes 2 main types of hypoperfusion with distinct therapeutic implications: distal embolic territorial hypoperfusion, which may respond to intra‐arterial thrombolysis, and ischemic core hypoperfusion (no‐reflow), which reflects microvascular injury and likely requires specific strategies targeting the neurovascular unit and preventively ideally applied prior to recanalization for optimal benefit. The variable association and mechanistic heterogeneity between these 2 hypoperfusion subtypes may account for the inconsistent efficacy of intra‐arterial thrombolysis reported in clinical trials. Standardized imaging protocols and timely identification of hypoperfusion will be essential to enhance mechanistic understanding, improve risk stratification and guide individualized treatment, thereby bridging the gap between angiographic recanalization and tissue‐level reperfusion.

## Author Contributions

Y.Q., and A.T.S. contributed to the conception and design of the manuscript; Y.Q., A.T.S., Y.X., J.‐C.B., and W.Z. contributed to the interpretation of studies included in the manuscript; and Y.Q., A.T.S., Y.X., J.‐C.B., and W.Z. contributed to drafting the text and preparing the figures. [Correction added on 25 February 2026, after first online publication: Author contribution text has been revised in this version.]

## Potential Conflicts of Interest

Nothing to report.

## Supporting information


**Supplementary Table S1.** Preclinical research on therapeutic targets for the no‐reflow phenomenon.

## Data Availability

Not applicable.
