# Supplementary material for: Revisiting Incomplete Tissue‐Level Reperfusion Following Successful Thrombectomy for Acute Ischemic Stroke
Source: Ann Neurol. 2026 Jan 26;99(3):668–83. doi: 10.1002/ana.78142 (PMC12954150; doi:10.1002/ana.78142)
Supplement: Supplementary file 1 — Supplementary Table S1. Preclinical research on therapeutic targets for the no‐reflow phenomenon. [file ANA-99-668-s001.docx]

**SUPPLEMENTAL TABLE**

**Table S1. Preclinical Research on Therapeutic Targets for the No-Reflow Phenomenon**

| Therapeutic Target | Agent or method | Timing | Approach | Effect | Animal | Model | Assessment of No reflow | References |
| --- | --- | --- | --- | --- | --- | --- | --- | --- |
| Microvascular obstruction | Anti-E-selectin monoclonal antibody | Pre- and postischemia (90 min) | Blocking E-selectin with monoclonal antibody to assess impact on ischemic injury and neutrophil accumulation | Increased cerebral blood flow (2.6-fold), reduced infarct volume, decreased neurological deficit | C57BL/6 mice | tMCAO/R (intraluminal suture occlusion, reperfusion after 45 min) | In vivo laser-Doppler probe | Huang et al., 2000^1^ |
|  | TP9201 | Pre-ischemia | Inhibition of integrin ɑIIbβ3 | Inhibition of platelet aggregation | Adolescent male baboons | tMCAO/R (180 min ischemia, 60 min reperfusion after balloon deflation) | Ex vivo section | Abumiya et al., 2000^2^ |
|  | PPARs activator (Fenofibrate) | Pre-ischemia (7 days) | Block pro-inflammatory IL-6 protein expression, and alleviate neuroinflammation, and apoptosis | Enhance cerebral blood flow recovery | C57BL/6 mice, male | tMCAO/R (120 min ischemia, filament withdrawal for reperfusion) | In vivo laser speckle imaging | Guo et al., 2010^3^ |
|  | Cilostazol | Pre-ischemia (7 days) | Decrease the expression of P-selectin and intercellular adhesion molecule-1 | Prevent platelet aggregation and leukocyte plugging in the microvessels | C57BL/6 J mice, male | tMCAO/R (45-90 min ischemia, filament withdrawal for reperfusion) | In vivo laser speckle imaging | Hase et al., 2012^4^ |
|  | Anti-Ly6G antibody | Pre-reperfusion (24 hours before imaging) | Clearance of neutrophils adhered to distal capillary segments | Reduce the infarct area and minimize neurological deficits. | BALB/c mice, male | Thrombin dMCAO (tissue plasminogen activator administered 30 min after ischemia) | In vivo laser speckle imaging, optical coherence tomography, and two-photon imaging | El Amki et al., 2020^5^ |
|  | Tongxinluo capsule | At 1.5 h, 22 h, 48 h, and 70 h post-ischemia | Suppress leukocyte-endothelial cell  Interactions and inhibit the expression of multiple inflammatory mediators | Improve neurological function, protect neurons, alleviate no-reflow | C57BL/6J mice, male | tMCAO/R (1.5h ischemia, filament withdrawal for reperfusion) | In vivo laser speckle imaging, two-photon microscopy | Liu et al., 2023^6^ |
| Pericyte Constriction | Superoxide scavengers  and NOS inhibitors | Pre-reperfusion (120 min) | Relieve pericyte contraction | Restored microvessels patency | C57/BL6 mice | tMCAO/R (120 min ischemia, filament withdrawal for reperfusion) | Ex vivo section | Yemisci et al., 2009^7^ |
|  | Iptakalim | In vivo: post-ischemia (1 h)  In vitro: post-ischemia (6 h) | Enhancement of the K-ATP  channels opening via suppressing SUR2/EPAC1 complex formation, relieve pericyte contraction | improve microvascular  disturbance | C57BL/6J mice, male | tMCAO (1 h ischemia/reperfusion)  OGD/R in vitro | In vivo laser speckle imaging and Ex vivo section | Guo et al., 2022^8^ |
|  | Fasudil (Rho kinase inhibitor) | Pre-ischemia | Relieve capillary pericyte contraction and release capillary stall | improve microvascular  disturbance | C57BL/6J mice, male; FACS -based isolation of pericytes | tMCAO/R (70 min ischemia, filament withdrawal for reperfusion) | In vivo laser speckle imaging and two-photon microscopy, laser Doppler fluxmetry (for ex vivo experiments) | Shrouder et al., 2023^9^​ |
| Endothelial Edema | Squalenoyl  adenosine Nanoparticle | Pre-ischemia or 2 h post-ischemia | Reduce erythrocyte entrapment and pericyte construction, alleviate endothelial cell and astrocyte edema. | Improve microcirculation via interaction with neurovascular unit | SWISS albino mice, male | tMCAO/R (120 min ischemia, filament withdrawal for reperfusion) | Ex vivo section | Gaudin et al., 2014^10^ |
| Astrocyte End Feet Edema | Transarterial regional  hypothermia | Pre-reperfusion (15 min) | Inhibition of the acute AQP4 surge | Attenuate microvascular narrowing, blood-brain barrier disruption, and  activation of other inflammatory reactions | Sprague-Dawley rats, male | tMCAO/R (120 min ischemia, filament withdrawal for reperfusion) | Ex vivo section | Kurisu et al., 2016^11^ |
|  | TRPV4 KO | - | Ameliorate astrocyte end-foot swelling | Preserve microcirculation and BBB integrity, reduce post-ischemic brain injury | C57BL/6N mice, male | tMCAO/R (30 min ischemia, filament withdrawal for reperfusion) | Ex vivo section | Tanaka et al., 2020^12^ |
| Increased vasoconstriction | ME-Linker overexpression | - | Restore myogenic spontaneous vasomotion | Accelerate reperfusion and improved neurological function | C57BL/6J mice, male and female | tMCAO/R (2h ischemia, filament withdrawal for reperfusion), primary cultured smooth muscle cells | In vivo imaging with two-photon microscopy, Laser Doppler flowmetry, laser speckle imaging | Li et al., 2024^13^ |
| Inflammation | Phosphodiesterase 10A | Immediately post- reperfusion | Inhibition of PDE10A, activate pro-survival pathways (Akt, Erk-1/2), suppress inflammatory cytokines | Improved neurological deficit scores, reduced infarct volume, improve cerebral microcirculation | Adult C57BL/6 mice, male | tMCAO/R (90 or 30 min ischemia, filament withdrawal for reperfusion) | In vivo laser speckle contrast imaging | Beker et al., 2021^14^ |
| Reactive Oxygen Species | Ebselen | Pre-ischemia (90 min) | Simulation of glutathione peroxidase, limited the induction and activation of MMP-9 | attenuated the increases in infarct size and vascular permeability | C57BL/6J and Gpx1-/- mice | tMCAO/R (2h ischemia, filament withdrawal for reperfusion) | In vivo laser Doppler flowmeter and fluorescence microscopy | Wong et al., 2008^15^ |
|  | vascular ROS suppression | shortly pre-reperfusion | Inhibit ROS formation in vascular walls | Reduced ischemic area, improve microcirculatory reperfusion. | Swiss mice, male | tdMCAO/R (1-hour ischemia, retract the micropipette for recanalization) | In vivo laser speckle imaging and Ex vivo  section | Taskiran-Sag et al., 2018^16^ |
| Inflammation and ROS | Astragalus | 2 h post-ischemia | Down-regulate JNK3 expression to inhibit neuronal apoptosis, reduce inflammation and ROS | Reduce infarct volume and improve neurological function | Wistar rats, male | tMCAO/R (2h ischemia, filament withdrawal for reperfusion) | In vivo laser speckle imaging | Liu et al., 2013^17^ |

Abbreviations: AQP4, Aquaporin-4; BBB, Blood-brain Barrier; dMCAO, Distal Middle Cerebral Artery Occlusion; FACS, Fluorescence-Activated Cell Sorting; Gpx1-/-, Glutathione Peroxidase 1 Knockout; IL-6, Interleukin-6; JNK3, c-Jun N-terminal Kinase 3; K-ATP, ATP-sensitive Potassium Channels; MMP-9, Matrix Metalloproteinase-9; NOS, Nitric Oxide Synthase; OGD/R, Oxygen-Glucose Deprivation/Reperfusion; PDE10A, Phosphodiesterase 10A; PPARs, Peroxisome Proliferator-Activated Receptors; ROS, Reactive Oxygen Species; SUR2/EPAC1, Sulfonylurea Receptor 2/Exchange Protein Directly Activated by cAMP 1; tMCAO/R, Transient Middle Cerebral Artery Occlusion/Reperfusion; TRPV4, Transient Receptor Potential Vanilloid 4.

**REFERENCES**

1. Huang J, Choudhri TF, Winfree CJ, McTaggart RA, Kiss S, Mocco J, et al. Postischemic cerebrovascular E-selectin expression mediates tissue injury in murine stroke. Stroke 2000;31:3047-3053.

2. Abumiya T, Fitridge R, Mazur C, Copeland BR, Koziol JA, Tschopp JF, et al. Integrin alpha(IIb)beta(3) inhibitor preserves microvascular patency in experimental acute focal cerebral ischemia. Stroke 2000;31:1402-1409; discussion 1409-1410.

3. Guo Q, Wang G, Namura S. Fenofibrate improves cerebral blood flow after middle cerebral artery occlusion in mice. J Cereb Blood Flow Metab 2010;30:70-78.

4. Hase Y, Okamoto Y, Fujita Y, Kitamura A, Nakabayashi H, Ito H, et al. Cilostazol, a phosphodiesterase inhibitor, prevents no-reflow and hemorrhage in mice with focal cerebral ischemia. Exp Neurol 2012;233:523-533.

5. El Amki M, Glück C, Binder N, Middleham W, Wyss MT, Weiss T, et al. Neutrophils Obstructing Brain Capillaries Are a Major Cause of No-Reflow in Ischemic Stroke. Cell Rep 2020;33:108260.

6. Liu S, Zhang Z, He Y, Kong L, Jin Q, Qi X, et al. Inhibiting leukocyte-endothelial cell interactions by Chinese medicine Tongxinluo capsule alleviates no-reflow after arterial recanalization in ischemic stroke. CNS Neurosci Ther 2023;29:3014-3030.

7. Yemisci M, Gursoy-Ozdemir Y, Vural A, Can A, Topalkara K, Dalkara T. Pericyte contraction induced by oxidative-nitrative stress impairs capillary reflow despite successful opening of an occluded cerebral artery. Nat Med 2009;15:1031-1037.

8. Guo RB, Dong YF, Yin Z, Cai ZY, Yang J, Ji J, et al. Iptakalim improves cerebral microcirculation in mice after ischemic stroke by inhibiting pericyte contraction. Acta Pharmacol Sin 2022;43:1349-1359.

9. Shrouder JJ, Calandra GM, Filser S, Varga DP, Besson-Girard S, Mamrak U, et al. Continued dysfunction of capillary pericytes promotes no-reflow after experimental stroke in vivo. Brain 2024;147:1057-1074.

10. Gaudin A, Yemisci M, Eroglu H, Lepetre-Mouelhi S, Turkoglu OF, Dönmez-Demir B, et al. Squalenoyl adenosine nanoparticles provide neuroprotection after stroke and spinal cord injury. Nat Nanotechnol 2014;9:1054-1062.

11. Kurisu K, Abumiya T, Nakamura H, Shimbo D, Shichinohe H, Nakayama N, et al. Transarterial Regional Brain Hypothermia Inhibits Acute Aquaporin-4 Surge and Sequential Microvascular Events in Ischemia/Reperfusion Injury. Neurosurgery 2016;79:125-134.

12. Tanaka K, Matsumoto S, Yamada T, Yamasaki R, Suzuki M, Kido MA, et al. Reduced Post-ischemic Brain Injury in Transient Receptor Potential Vanilloid 4 Knockout Mice. Front Neurosci 2020;14:453.

13. Li J, Zhang Y, Zhang D, Wang W, Xie H, Ruan J, et al. Ca(2+) oscillation in vascular smooth muscle cells control myogenic spontaneous vasomotion and counteract post-ischemic no-reflow. Commun Biol 2024;7:332.

14. Beker MC, Caglayan AB, Altunay S, Ozbay E, Ates N, Kelestemur T, et al. Phosphodiesterase 10A Is a Critical Target for Neuroprotection in a Mouse Model of Ischemic Stroke. Mol Neurobiol 2022;59:574-589.

15. Wong CH, Bozinovski S, Hertzog PJ, Hickey MJ, Crack PJ. Absence of glutathione peroxidase-1 exacerbates cerebral ischemia-reperfusion injury by reducing post-ischemic microvascular perfusion. J Neurochem 2008;107:241-252.

16. Taskiran-Sag A, Yemisci M, Gursoy-Ozdemir Y, Erdener SE, Karatas H, Yuce D, et al. Improving Microcirculatory Reperfusion Reduces Parenchymal Oxygen Radical Formation and Provides Neuroprotection. Stroke 2018;49:1267-1275.

17. Liu G, Song J, Guo Y, Wang T, Zhou Z. Astragalus injection protects cerebral ischemic injury by inhibiting neuronal apoptosis and the expression of JNK3 after cerebral ischemia reperfusion in rats. Behav Brain Funct 2013;9:36.
